# Midwifery Qualification in Selected Countries: A Rapid Review

**DOI:** 10.3390/nursrep11040080

**Published:** 2021-10-26

**Authors:** Shakirah Md. Sharif, Wuan Shuen Yap, Weng Hong Fun, Ee Ling Yoon, Nur Fadzilah Abd Razak, Sondi Sararaks, Shaun Wen Huey Lee

**Affiliations:** 1 Institute for Health Systems Research, National Institutes of Health, Ministry of Health Malaysia, Shah Alam 40170, Malaysia; sereneyap07@gmail.com (W.S.Y.); fun.wh@moh.gov.my (W.H.F.); eelingyoon@gmail.com (E.L.Y.); fadzilahrazak89@gmail.com (N.F.A.R.); sararaks.s@moh.gov.my (S.S.); 2 School of Pharmacy, Monash University, Bandar Sunway 47500, Malaysia; shaun.lee@monash.edu

**Keywords:** midwifery qualification, midwifery practice, rapid review

## Abstract

Background: While the global maternal mortality ratio (MMR) shows a decreasing trend, there is room for improvement. Midwifery education has been under scrutiny to ensure that graduates acquire knowledge and skills relevant to the local context. Objective: To review the basic professional midwifery qualification and pre-practice requirements in countries with lower MMR compared with Malaysia. Methods: A rapid review of country-specific Ministry of Health and Midwifery Association websites and Advanced Google using standardised key words. English-language documents reporting the qualifications of midwives or other requirements to practise midwifery from countries with a lower MMR than Malaysia were included. Results: Sixty-three documents from 35 countries were included. The minimum qualification required to become a midwife was a bachelor’s degree. Most countries require registration or licensing to practise, and 35.5% have implemented preregistration national midwifery examinations. In addition, 13 countries require midwives to have nursing backgrounds. Conclusion: In countries achieving better maternal outcomes than Malaysia, midwifes often have a degree or higher qualification. As such, there is a need to reinvestigate and revise the midwifery qualification requirements in Malaysia.

## 1. Introduction

Globally, there has been improvement in the number of maternal deaths over a span of 25 years, due to better healthcare access and improvements in quality of care [1]. Similarly, Malaysia has recorded an overall decrease in the maternal mortality rate (MMR) per 100,000 live births, which fell by 24% between 2000 and 2017, but this trend has plateaued from 2006 onwards [2]. The latest World Bank statistics in 2017 reported Malaysia’s MMR at 29 per 100,000 live births [3]. Although this number is below the global target set in the Sustainable Development Goal Target 3.1, Malaysia is aiming to further lower its MMR to the single digit range, as maternal deaths are often preventable and reflect the quality of maternal care [2,4]. Areas such as effective coordination and communication among providers for pregnant women, early identification, diagnosis and treatment of women with “high risk” status could influence maternal mortality rates [5,6,7]. Without sufficient coverage, access and support from maternity care providers especially midwives, this possesses great barriers in preventing avoidable maternal deaths [8].

One of the established strategies is to improve access to skilled midwifery practitioners and provide adequate health facilities, especially as it could significantly reduce maternal mortality and morbidity [9]. For example, Uganda has recently upgraded its midwifery education to a Bachelor’s degree, and enabled midwives to employ higher-level skills compared to their certificate-trained counterparts [10]. To strengthen health systems’ responses to maternal and child health, WHO recommends workforce management with regulated care providers as well as education and core competencies that meet global standards [11]. These standards can be found in the Global Standards for Midwifery Education Amended 2013, which has listed the minimum expectations for a midwifery programme, focusing on education that emphasises competency as a measure of quality assurance [12].

Historically, traditional birth attendants provided midwifery care in Malaysia. Access to skilled maternal care in Malaysia was expanded with community nurses providing basic maternal and child health (MCH) services in the public system [13], where the majority of service-utilisation is seen [14]. Trained through a two-and-a-half-year course at Ministry of Health Training Institutes, they serve rural community clinics where they are forefront health care providers, or MCHC clinics with a team comprising registered nurses and doctors [15]. In 2018, the ratio of community nurses per capita was 1 to 1379 [16]. Although the training of new community nurses ceased in 2013 [17], community nurses are still being used to deliver maternal and child health care. Nurse–midwives with one-year post-nursing midwifery training or public health nurses also provide midwifery services at primary care level [18].

An expectant mother in Malaysia with an uncomplicated pregnancy has at least seven visits to midwives throughout the antenatal period, and two routine visits where they are seen by the doctor. At each visit to the midwife, routine blood pressure, urine screening and an antenatal check-up are carried out, from which any abnormality discovered will be referred to the doctor. Community nurses manage only uncomplicated pregnancies while nurse–midwives manage the rest, in close partnership with doctors. During the postnatal period, community nurses and nurse–midwives conduct home visits to ensure the well-being of mother and baby [19].

Shortcomings in the management of obstetric complications are postulated to be contributing factors towards maternal mortality in Malaysia [20], similar to patterns seen globally [4]. The stagnant MMR in Malaysia has raised concerns among maternal health policymakers on the competency and skills of certificate- or diploma-qualified primary care nurses in managing increasingly complex maternal cases, as they require the skills to identify early indicators of risk and refer appropriately in a timely manner. It has been acknowledged that provision of maternal care by skilled providers, “trained, educated, licensed and regulated midwives” [21], can have a profound effect on maternal outcomes. Therefore, this study aims to review the basic qualifications and other requirements to practise as a recognised midwife in countries with better MMRs than Malaysia.

## 2. Materials and Methods

A rapid document review was conducted using streamlined systematic review methods [22], and reported using the Preferred Reporting Items for Systematic Reviews and Meta-Analysis (PRISMA) guidelines [23]. The protocol was registered with Open Science Framework [24].

### 2.1. Study Search

We searched PUBMED, CINAHL, EMBASE and Health Systems Evidence for studies published between 2000 to 2019. However, due to the absence of relevant articles, two reviewers (YEL and NFAR) conducted handsearching of relevant websites and Google Advanced Search using standardised key words in March 2019 [25]. Details on the search strategy can be found in Appendix A.

### 2.2. Study Inclusion

Studies were selected if they were: (1) documents from countries with a reported MMR lower than Malaysia’s based on the 2015 World Bank data (any country with MMR < 40); (2) documents from credible sources such as law documents, journal articles, articles, assessment reports, theses (doctoral), association reports, midwifery association reports, midwifery association web pages, government policies, standards, government web pages, government agencies circulars and documents; (3) documents available in English; (4) documents on the qualifications of lawfully recognised midwives or requirements to practise. Any qualification that rendered its holder able to provide midwifery services legally, with or without the professional title of “midwife”, was included.

### 2.3. Study Selection, Quality Assessment and Data Extraction

Relevant study titles were screened for eligibility independently by two reviewers (YEL, NFAR), and potentially relevant studies were retrieved. Any uncertainties during screening were discussed with a third reviewer (SMS). Data were extracted independently by two reviewers (YEL, NFAR) using a predesigned and piloted data extraction table for synthesis. Verification was conducted by the principal investigator (SMS) on 10% of randomly selected records from each reviewer. Quality assessment using the Authority, Accuracy, Coverage, Objectivity, Date and Significance (AACODS) checklist [26] was completed by YEL and NFAR.

The review was registered in the National Medical Research Registry (NMRR-18-3421-45549) and exempted from the Medical Research Ethics Committee (KKM/NIHSEC/P19-1528 (4)).

### 2.4. Analysis

A narrative synthesis of the included documents was performed, followed by a comparison between the qualification levels of midwifery practitioners in Malaysia and those in the included countries. Additionally, we looked at other requirements to be fulfilled in order to practise as a recognised midwife in the included countries.

## 3. Results

### 3.1. Overview of Included Documents

**Figure 1:**
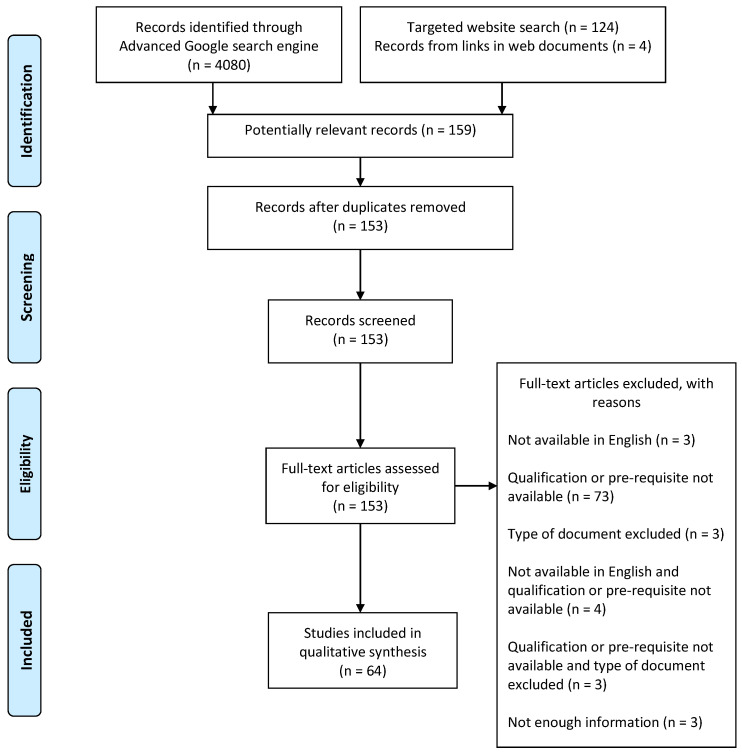
PRISMA flow diagram of the review.

The search identified 4204 records, with 124 derived from handsearching of websites and 4080 from Advanced Google (Figure 1). A total of 153 records were further identified and screened for eligibility, and 64 documents were included in this rapid review. The study flow is shown in Figure 1.

These 64 documents described the qualifications to practice midwifery from 35 countries (Table 1). The majority of the included information was from midwifery governing body web pages (*n* = 11) [27,28,29,30,31,32,33,34,35,36,37] and law documents (*n* = 11) [38,39,40,41,42,43,44,45,46,47,48] followed by midwifery assessment reports (*n* = 10) [49,50,51,52,53,54,55,56,57,58] and midwifery association web pages (*n* = 8) [59,60,61,62,63,64,65,66]. The rest of the documents were from published articles (*n* = 7) [67,68,69,70,71,72,73], government web pages (*n* = 7) [74,75,76,77,78,79,80], practice standards (*n* = 5) [81,82,83,84,85], government documents (*n* = 4) [86,87,88,89] and a doctoral thesis (*n* = 1) [90]. Quality appraisal using the AACODS checklist ranged from 4 to 6.

### 3.2. The Minimum Midwifery Qualification

The minimum qualification for midwives in the included documents ranged from a certificate to a master’s degree. The most common lowest qualification was a direct-entry degree in midwifery (46%, *n* = 16) [28,30,33,40,43,53,54,60,61,67,69,72,74,75,81,82,84,86,87,89,91]. Three countries (9%) required nursing degree-holders with a postgraduate-level midwifery qualification [38,49,68], whereas another two (6%) required midwifery qualification at the master’s level [64,90]. Similar to Malaysia, four countries (11%) were found to require midwives to possess a diploma in nursing supplemented by a post-nursing qualification in midwifery [32,39,50,52,57,59], whereas another four (11%) had certificate-level midwives (Table 1) [45,48,58,73].

**Figure 2:**
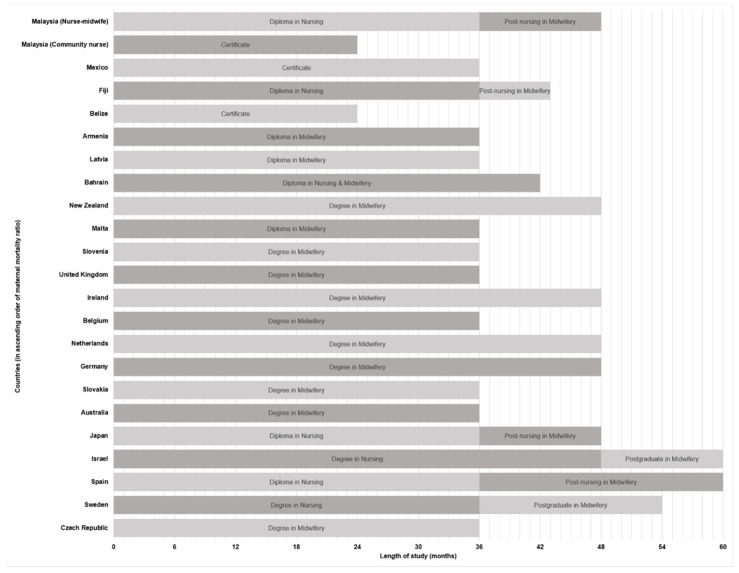
Study duration to become a midwife.

Among the included countries with complete information on study duration (*n* = 21), it was observed that most direct-entry degrees (*n* = 10) [31,33,53,54,60,61,67,69,70,75,84,87,91] or diplomas (*n* = 3) [41,44,56] in midwifery were three- or four-year-long courses, whereas post-nursing (*n* = 3) [39,50,51,52,57,59] or postgraduate (*n* = 2) [49,68] midwifery qualifications required between seven months to two years of study (Figure 2).

### 3.3. Other Requirements to Practise as a Recognised Midwife

Requirements other than midwifery education were not available for four countries. Of the 31 countries with available information, 28 (90.3%) required registration and/or licensing in midwifery. Eleven (35.5%) implemented a preregistration national midwifery examination [33,35,39,40,42,45,47,52,55,59,62,65,68,69,71,77,78,88,89]. Additionally, 13 (41.9%) countries required midwives to have a nursing background [32,36,38,39,46,49,50,51,52,57,59,63,64,65,66,68,71,73,79,85,90]. In addition to nursing education, seven (22.6%) countries also required registration and/or licensing in nursing [36,49,57,64,65,68,71,79,90]. Only two countries (6.5%) required new midwives to undergo supervised in-service training [42,44]. Table 2 summarises this information.

## 4. Discussion

### 4.1. Higher-Educated Midwives Add Value to the Practice

Direct-entry midwifery degrees were found to be the minimum qualification in the majority of countries included. In comparison, most midwifery practitioners in Malaysia are community nurses [92] with certificate-level qualification, followed by nurses with basic qualification at certificate, diploma or degree level with a post-nursing advanced diploma in midwifery or public health [18]. The risk-approach system used in Malaysia [19] delineates case management by nurses of different qualification levels, where only low-risk cases are managed by community nurses and mild to moderate risk cases are managed by nurses.

Community nurses in Malaysia mainly serve rural clinics. While community mobilisation improves access to care, variable quality of care remains a challenge [93]. As such, WHO has called for midwives to be educated and trained to fulfil international standards, in an effort to strengthen midwifery education [94]. In Australia, recognising the need for multiskilled practitioners in the rural community, a four-year double degree programme combining nursing and midwifery was introduced in 2008 [95]. Iceland, with an MMR of three per 100,000 population in 2015 [3], have nurse–midwives, where only the best nursing students are able to qualify for a seat in midwifery training [96]. Therefore, revising the minimum qualification holds the potential for improving midwifery care standards. Additionally, further education of current community nurses can be enhanced to elevate them to the level of nurse–midwives. This would enable practise of the full scope of midwifery care as spelled out in the Framework for Action Strengthening Quality Midwifery Education for Universal Health Coverage 2030 [94].

A higher-qualified nursing workforce is valued for their variety of skills from critical thinking to effective health promotion across both inpatient and outpatient settings [97]. Undergraduate nurses were found to perform better in areas of professional practice compared to their lower-educated counterparts [98]. Various studies found that increasing the proportion of degree-qualified nurses among hospital staff resulted in lower in-patient mortality rates [99,100,101,102]. In the United States, there has been a shift to encourage nurses to obtain a bachelor’s degree to achieve the Institute of Medicine’s recommendation for more degree-level nurses, owing to evidence of better patient outcomes with degree-level nurses [103]. Although the available literature evaluating the impact of nurses’ qualification levels is hospital-focused, it can be postulated that having degree-level midwives in primary care will result in better patient outcomes. Moreover, the Chair of the Nursing and Midwifery Council in the United Kingdom highlighted the need to have degree-level education to meet increasing work demands [104].

A participant-assessed study of undergraduates and diplomates of nursing or midwifery found that undergraduates performed better in areas of cognitive ability and reflective practice ability [105]. Shin [106] evaluated critical thinking abilities of Korean senior nursing students and found that those enrolled in undergraduate programmes scored better than those in associate degree programmes. In solving complex problems, the ability to think critically is vital in enabling midwives to arrive at the best clinical decisions in a timely manner [107]. Current evidence linking critical thinking and clinical decision-making abilities in nursing is disputable, due to the uncertain validity of methods used in measuring critical thinking, but overall such evidence seems to be credible [108]. You et al. [109] highlighted that having better-qualified nurses is essential with the expansion of nurses’ roles in the community and the increasing complexity-level of care. In public healthcare systems that are often challenged by finite resources, employing undergraduate nurses who are trained to be critical thinkers and problem solvers under an evidence-based curriculum is valuable, as they are able to adapt and adjust their practice accordingly [110].

Undergraduate nurses are also more likely to be educators, participate in research and incorporate best evidence into practice [111]. Evidence-based practice (EBP), ”the conscientious, explicit, and judicious use of current best evidence in making decisions about the care of individual patients” [112] (p.71) positively impacts patient outcomes in nursing and midwifery, reduces healthcare costs and empowers nurses and midwives, resulting in the WHO Regional Office in Europe urging its Member States to encourage and develop EBP culture in nursing and midwifery [113]. An integrative review found that although EBP in midwifery is valued, implementation is still lacking [114]. Similarly, Lai et al. [115] found that nursing and allied health practitioners in several Malaysian hospitals reported less favourable attitudes towards EBP, which could possibly be due to the low confidence attributed to their diploma qualification level. This supports the need to upgrade the qualifications of all Malaysian midwives to degree level, with emphasis on evidence-based practice as a strategy to potentially improve all maternal outcomes as they often face clinical decision-making junctures in practice.

Higher education is an empowering tool for day-to-day practice in midwifery, as midwives are at the forefront of primary care and need to be not only prepared in providing consistent advice in antenatal care, but also equipped to skilfully promote family planning, a core strategy that reduces risk of maternal death [116]. Effective counselling on preconception care by nurses or midwives has the potential to assist a woman in preparing herself physically and mentally to sustain a pregnancy [117]. Women who receive preconception counselling are more inclined to improve their health behaviour [118] and lifestyle [119] before becoming pregnant. In recognition of the extensive duties expected of a competent midwife, having better-qualified midwives will facilitate quality improvement in maternal care.

The duration of study for most direct-entry midwifery qualifications is three or four years in the included countries, while the duration of post-nursing or postgraduate studies ranges between seven months to two years. In Malaysia, graduates with community nurse certificates were simultaneously trained in midwifery and basic nursing over the short span of two-and-a-half years [120]. While community nurses are only expected to provide care for low-risk pregnancies, they are required to identify signs of escalating pregnancy risk and recognise the need for referral, while failure to do so can put both mother and child in imminent danger. Hence, with increasingly complex maternal cases, it is important for community nurses to acquire adequate knowledge and skillsets to meet expanding demands. Since effective midwifery is projected to improve outcomes, [21,116] the situation in Malaysia raises questions on whether the curriculum of local midwifery programmes is comprehensive enough to produce graduates who are as competent as the midwives with longer training durations in countries with lower MMR.

### 4.2. Producing Work-Ready, Quality-Assured Midwives

A national midwifery examination and subsequent registration or licensing ensures all prospective midwives meet the same competency standards prior to practising. Malaysian midwifery students are required to pass the national midwifery examination mandated by the Midwifery Board of Malaysia (*Lembaga Bidan Malaysia*) to qualify for registration [121]. We found that 11 out of 31 countries with available information implemented a preregistration national midwifery examination, whereas nearly all the countries required midwives to obtain midwifery registration and/or licensing in order to practise legally.

Midwifery registration or licensing is a certification, a formal mechanism enforced to regulate quality and competency. Certification, defined as ”a process by which an authorised body, either a governmental or nongovernmental organisation, evaluates and recognises either an individual or an organisation as meeting predetermined requirements or criteria”, has long been accepted as a quality improvement strategy in various healthcare professions [122] (p. 3). In the Malaysian context, midwives are governed by the Midwifery Act, which requires every person practising midwifery to be registered [121]. A literature review on how nurses perceive specialty certification reported that certified nurses were more satisfied with their job, felt more empowered in their practice and had a greater sense of collaboration with other healthcare professionals. These nurses found that certification facilitated professional growth, and had proof that their abilities were on par with those of the practice standard [123]. Certified nurses also reported being better able to intervene and prevent adverse outcomes due to an increased ability to recognise changes in patient status [124]. In another study, it was found that surgical wards with more certified specialty nurses had a lower rate of central-line-associated bloodstream infections [125]. Certification was thought to enhance nurses’ autonomy in practice and clinical expertise, consequently improving patient outcomes [123]. Additionally, certification allows the patient to trust in the abilities of the healthcare professional in managing their health [126]. Hence, certification by a regulatory body validates the professional autonomy of a midwife, consequently generating confidence among their clients.

”Learning through doing” [127] is crucial in preparing graduates for a practical profession like midwifery. This is reflected in the recommendation by the International Confederation of Midwives, where it is stated in the Global Standards for Midwifery Education that, “the midwifery curriculum should include both theory and practise elements with a minimum of 40% theory and a minimum of 50% practise” [12] (p. 6). Our search did not reveal the actual extent of practical skills and experience required of midwifery students upon completion of their studies. However, concerns pertaining to the sufficiency of clinical preparation of midwifery graduates have been voiced [128,129]. Graduates have described the transition from student to qualified, professional midwife as challenging [130], stressful, an unexpected reality [131] and overwhelming [132], due to a perceived lack of knowledge and experience.

Two countries (Singapore and Latvia) reported the requirement of supervised in-service training for new midwives in our study. In Malaysia, new nurses and midwives are supported via an unofficial mentor–mentee programme where the mechanics are not set, and may differ in execution [17,133]. In contrast, New Zealand runs a compulsory midwifery transition programme known as the “Midwifery First Year of Practice” (MYFP) programme aimed to support new midwives in building confidence as independent practitioners, using a mentor–mentee approach with continuous professional development [134]. The programme has also been found to boost retention regardless of age, race, level of education or place of work [135]. A study of midwifery students in Turkey found that internships and night shift practical training were perceived to be beneficial in preparing students for professional practice [136]. Similarly, a study of Irish midwifery students who undergo a paid 36-week internship in final year found students were able to apply knowledge into real-world practice which in turn builds confidence [127]. In Norway, a comparison between midwives who had a one-year internship as part of their two-year course and midwives who did not, found that the latter felt less prepared for practice than the former [128]. Evidently, be it internships or transition programmes, supervised clinical training prepares midwifery students in becoming self-efficacious practitioners.

### 4.3. Limitations

Although strengthening midwifery education has the potential to reduce maternal mortality, there are other associated factors that must be considered in order to improve maternal outcomes.

The database search did not yield any relevant documents that related midwifery qualifications to maternal outcomes. As such, a grey literature search was carried out. Although every effort was taken to retrieve the latest country-specific documents, there may be a delay in availability of document updates online, and thus the documents may not reflect the current country practice. Some countries may label a qualification as a “diploma”, but it may be equivalent to a degree or higher. Additionally, the prerequisites and duration of study for the qualification should be considered. Not all the included countries in this review had complete information, nor were we able to retrieve information from all the countries identified as having a lower MMR than Malaysia.

While some countries offered multiple pathways into midwifery, this study only looked at the minimum levels of qualification required to practise as a midwife. The proportion of midwives corresponding to different qualification levels in each country was not considered. Also, we only included government-recognised midwifery qualifications. Authors or relevant midwifery organisations in other countries were not contacted for further information in this review. When required, permission was sought to use information from the included documents.

This study provides a comparison between Malaysia and countries with lower MMR than Malaysia. It would also be helpful to make comparisons with countries with higher MMR in the future to gain a comprehensive comparative understanding of global midwifery education.

## 5. Conclusions

Most countries achieving better maternal mortality outcomes than Malaysia had degree-level midwives. Revision of the qualification requirements of midwives has significant potential for improving maternal care quality and hence reducing MMR. In order to produce midwives who can stand independently at the forefront of increasingly complex maternal health demands, areas for improvement in their education must continue to be identified and addressed to ensure their continuing competency and professional development meets international standards.

## Figures and Tables

**Table 1:** Minimum qualification required to practise as a recognised midwife.

Source	Title	Type of Document	Document ID	Country	Qualification	MMR
Ministry of Welfare, 2012 [38]	Regulation on the education, rights and obligations of midwives and criteria for granting of licences and specialist licences, No. 1089/2012	Law document	44	Iceland	Degree in Nursing + Postgraduate in Midwifery	3
Urbánková et al., 2018 [67]	The Assessment of the Quality of Human Resources in the Midwife Profession in the Healthcare Sector of the Czech Republic	Published article	10	Czech Republic	Degree in Midwifery	4
Emons & Luiten, 2001 [49]	Midwifery in Europe: An inventory in fifteen EU-member states	Assessment report	26	Sweden	Degree in Nursing + Postgraduate in Midwifery	4
Weiss, et al., 2017 [86]	Healthcare Professions in Austria 2017	Government document	50	Austria	Degree in Midwifery	4
Emons & Luiten, 2001 * [49]	Midwifery in Europe: An inventory in fifteen EU-member states	Assessment report	26			
Ruiz-Berdún, 2016 [50]	Chapter 1: The competences of midwives in Spain over time in Maternity care in different countries Midwife’s contribution	Assessment report	4	Spain	Diploma in Nursing + Post-nursing in Midwifery	5
Leon-Larios, 2016 [51]	Chapter 2: The current situation of midwives in Spain in Maternity care in different countries: Midwife’s contribution	Assessment report	4			
Natan & Ehrenfeld, 2011 [68]	Nursing and midwifery education, practice, and issues in Israel	Published article	14	Israel	Degree in Nursing +Postgraduate in Midwifery	5
Japanese Nursing Association, 2018 [52]	Midwifery in Japan	Assessment report	25	Japan ^a^	Diploma in Nursing + Post-nursing in Midwifery	5
Act on Public Health Nurses, Midwives, and Nurses, Act No.2013 1948 [39]	Act on Public Health Nurses, Midwives, and Nurses, Act No.2013 1948	Law document	38			
Public Interest Incorporated Foundation [59]	Japanese Society of Midwifery Education: How to become a midwife in Japan	Midwifery association web page	63			
Nursing and Midwifery Board of Australia, 2017 [27]	Registration Standards	Governing body web page	15	Australia	Degree in Midwifery	6
Australian Nursing and Midwifery Accreditation Council, 2014 [28]	Midwife Accreditation Standards 2014	Governing body web page	29			
Australian College of Midwives, 2019 [60]	Is Midwifery for Me?	Midwifery association web page	59			
Beňušová et al., 2006 [87]	Education of healthcare professionals in the Slovak Republic	Government document	21	Slovakia	Degree in Midwifery	6
UAE Nursing and Midwifery Council, 2013 [81]	UAE Nursing and Midwifery Education Standards	Standards	47	United Arab Emirates	Degree in Midwifery	6
Mattern, et al., 2017 [69]	Experiences and wishes of women regarding systemic aspects of midwifery care in Germany: a qualitative study with focus groups	Published article	45	Germany	Degree in Midwifery	6
Emons & Luiten, 2001 * [49]	Midwifery in Europe: An inventory in fifteen EU-member states	Assessment report	26			
Zondag, et al., 2017 [91]	Midwifery in the Netherlands 2017	Assessment report	37	Netherlands ^b^	Degree in Midwifery	7
Emons & Luiten, 2001 * [49]	Midwifery in Europe: An inventory in fifteen EU-member states	Assessment report	26			
Federal Public Service (FPS) Health, Food Chain Safety and Environment, 2016 [74]	Regulated healthcare professions in Belgium	Government web page	64	Belgium	Degree in Midwifery	7
Federal Public Service (FPS) Health, Food Chain Safety and Environment, 2016 [80]	Healthcare providers	Government web page	41			
Emons & Luiten, 2001 * [49]	Midwifery in Europe: An inventory in fifteen EU-member states	Assessment report	26			
Beeckman K., & Reyns M., 2016 ** [53]	Chapter 6: Midwifery in Belgium in Midwifery Care in Different Countries	Assessment report	4			
Nursing and Midwifery Board of Ireland, 2005 [82]	Requirements and Standards for the Midwife Registration Education Programme Third Edition	Standards	17	Ireland	Degree in Midwifery	8
Nursing and Midwifery Board of Ireland [29]	Registration	Governing body web page	18			
Nursing and Midwifery Board of Ireland [30]	Careers in Nursing and Midwifery: Where to study to become a nurse or midwife?	Governing body web page	19			
Nursing and Midwifery Board of Ireland [83]	Midwife Post-RGN Registration: Standards and requirements	Standards	20			
Emons & Luiten, 2001 * [49]	Midwifery in Europe: An inventory in fifteen EU-member states	Assessment report	26			
Daly D., 2016 ** [54]	Chapter 5: The maternity services and becoming a midwife in Ireland in Midwifery Care in Different Countries	Assessment report	4			
Gasser et al., 2008 [55]	The profession of midwives in Croatia	Assessment report	60	Croatia	Degree in Midwifery	8
Midwifery Act, 2008 [40]	Midwifery Act (consolidated text, OG 120/08, 145/10)	Law document	27			
The Royal College of Midwives [61]	How to become a midwife?	Midwifery association web page	2	United Kingdom	Degree in Midwifery	9
Nursing and Midwifery Council [84]	Standards for competence for registered midwives	Standards	5			
Nursing and Midwifery Council, 2019 [31]	Becoming a midwife	Governing body web page	51			
Ministry of Health, 2017 [75]	Licence for independent practice of nursing and midwifery services	Government web page	33	Slovenia	Degree in Midwifery	9
Ministry of Health, 2017 [76]	Entry in the register of nursing and midwifery service	Government web page	46			
Mivšek et al., 2015 [70]	How do midwives in Slovenia view their professional status?	Published article	40			
Health Care Professions Act, Chapter 464, 2003 [41]	Health Care Professions Act, Chapter 464, 2003	Law document	43	Malta	Diploma in Midwifery	9
Nurses and Midwives Act Chapter 209, 2012 Revised Edition [42]	Nurses and Midwives Act Chapter 209, 2012 Revised Edition	Law document	16	Singapore	Diploma in Nursing + Post-nursing in Midwifery	10
Singapore Nursing Board, 2019 [32]	Local graduates	Governing body web page	32			
Midwifery Council of New Zealand [33]	What does it take to qualify as a midwife?	Governing body web page	1	New Zealand	Degree in Midwifery	11
New Zealand College of Midwives [62]	Regulation	Midwifery association web page	8			
Midwifery Council of New Zealand [34]	Maintaining competence	Governing body web page	55			
Midwifery Council of New Zealand [35]	Becoming Registered to Practise	Governing body web page	65			
Lee, 2003 [71]	Improving the Standards of Midwifery Education and Practice and Extending the Role of a Midwife in Korean Women and Children’s Health Care	Published article	39	Korea	Unspecified Nursing Education + Midwifery Education	11
Jahlan, 2016 [90]	Perspectives on Birthing Services in Saudi Arabia	Doctoral thesis	9	Saudi Arabia	Degree in Nursing + Master’s in Midwifery	12
American College of Nurse-Midwives, 2011 [63]	Definition of Midwifery and Scope of Practice of Certified Nurse–Midwives and Certified Midwives	Midwifery association web page	11	United States ^c^	Degree in Nursing + Master’s in Midwifery	13
American College of Nurse-Midwives [64]	Information for Midwives Educated Abroad	Midwifery association web page	23			
Midwifery Education and Accreditation Council, 2019 [36]	Frequently Asked Questions (FAQ)	Governing body web page	31			
American College of Nurse Midwives [65]	Comparison of Certified Nurse–Midwives, Certified Midwives, Certified Professional Midwives Clarifying the Distinctions Among Professional Midwifery Credentials in the US	Midwifery association web page	48			
American College of Midwives [66]	Become a Midwife	Midwifery association web page	52			
North American Registry of Midwives [37]	How to become a CPM	Governing body web page	56			
Qatar Council for Health Practitioners [88]	Nursing Regulations in the state of Qatar	Government document	12	Qatar	Degree in Midwifery	13
Qatar Council for Health Practitioners, 2016 [89]	Circular No. (12/2016)	Government document	49			
Ministry of Public Health, 2017 [77]	Healthcare Practitioners Registration & Licensing	Government web page	57			
Ministry of Public Health, 2018 [78]	Information about the Qatar Council for Health Practitioners (QCHP)	Government web page	67			
Lebanese Order of Midwives, 2018 [43]	Lebanese Order of Midwives, 2018	Law document	34	Lebanon	Degree in Midwifery	15
National Health Regulatory Authority, 2017 [85]	Healthcare Professional Licensing Standards: Nurses 2017	Standards	42	Bahrain ^d^	Diploma in Nursing & Midwifery	15
Law on Regulated Professions and Recognition of Professional Qualifications, 2001 [44]	Law on Regulated Professions and Recognition of Professional Qualifications, 2001	Law document	36	Latvia	Diploma in Midwifery	18
Nursing and Midwifery Profession Act B.E. 2528, 1985 [45]	Nursing and Midwifery Profession Act B.E. 2528, 1985	Law document	13	Thailand	Certificate	20
Lillo et al., 2016 [72]	Midwifery in Chile, A Successful Experience to Improve Women’s Sexual and Reproductive Health: Facilitators & Challenges	Published article	54	Chile	Degree in Midwifery	22
Sahakayan et al., 2019 [56]	An Evaluation of Midwifery Education System in Armenia	Assessment report	53	Armenia	Diploma in Midwifery	25
Ministry of Health Government of Grenada, 2016 [79]	Careers in Nursing: Midwifery	Government web page	28	Grenada	Unspecified Nursing Education + Midwifery Education	27
Chapter 194 Midwives Act, 2003 [46]	Chapter 194 Midwives Act, 2004	Law document	35			
Boyer, 2001 [73]	Midwifery in Northern Belize	Published article	24	Belize ^e^	Certificate	28
Nurses and Midwives Registration Act, Chapter 321, 2003 [47]	Nurses and Midwives Registration Act, Chapter 321, 2003	Law document	62			
Western Pacific Region Nursing and Midwifery Databank, 2013 [57]	Western Pacific Region Nursing and Midwifery Databank. Country: Fiji	Assessment report	61	Fiji	Diploma in Nursing + Post-nursing in Midwifery	30
Medical Ordinance, Chapter 113 [48]	Medical Ordinance, Chapter 113	Law document	30	Sri Lanka	Certificate	30
Atkin et al., 2017 [58]	Midwifery in Mexico	Assessment report	58	Mexico ^f^	Certificate	38

* Information from this document was used to supplement information for other countries (Austria, Germany, Netherlands, Belgium and Ireland) where applicable. ** Different chapters from the same document. ^a^ The qualification from the training school for nurses is considered to be equivalent to a diploma, whereas the qualification from the training school for midwives is considered to be a post-nursing qualification. Japan has numerous pathways to becoming a midwife, but this pathway was considered to be the minimum qualification required. ^b^ Primary care midwife considered here. ^c^ Although the United States offers numerous midwife qualifications, only Certified Nurse–Midwives (CNM) were considered here because CNMs are the most widely recognised, and are licensed to practise in most areas in the United States. ^d^ The General Nurse (Diploma in Nursing and Midwifery) qualification considered here. ^e^ The rural health nurse qualification is considered to be equivalent to a certificate. ^f^ The technical midwife qualification is considered to be equivalent to a certificate.

**Table 2:** Requirements to practise as a midwife.

Country		Maternal Mortality Ratio (MMR)	Requirements to Practise as a Recognised Midwife *								
			Nursing Education	National Nursing Examination	Registration as a Nurse	Licence in Nursing	Work Experience	Supervised In-Service Training	National Midwifery Examination	Registration as a Midwife	Licence in Midwifery
Iceland		3	●								●
Czech Republic		4	No information available								
Sweden		4	●		●		Nurse (6 months)				●
Austria		4								●	
Spain		5	●								
Israel		5	●	●	●				●		●
Japan ^a^		5	●	●					●	●	●
Australia		6								●	
Slovakia		6									●
United Arab Emirates		6								●	●
Germany		6							●	Not required	
Netherlands ^b^		7								●	
Belgium		7								●	●
Ireland		8								●	
Croatia		8							●	●	
United Kingdom		9								●	
Slovenia		9								●	●
Malta		9								●	●
Singapore		10	●					● **	●	●	● ***
New Zealand		11							●	●	
Korea		11	●			●			●		●
Saudi Arabia		12	●		●					●	
United States ^c^		13	●		●	●			●		●
Qatar		13							●	●	●
Lebanon		15								●	
Bahrain ^d^		15	●								
Latvia		18						●		●	
Thailand		20							●	●	●
Chile		22	No information available								
Armenia		25	No information available								
Grenada		27	●		●					●	●
Belize ^e^		28	●						●	●	●
Fiji		30	●		●		Nurse (2 years)			●	
Sri Lanka		30								●	
Mexico ^f^		38	No information available								
Malaysia	Community Nurse	40	●							●	
	Nurse-Midwife		●	●	●		Nurse (2 years)		●	●	

For countries with multiple pathways into midwifery, only the registration requirements for graduates of “purely midwifery” courses or a lower qualification that includes midwifery training were considered except in the United States, due to the different legislations governing midwifery in each * Other than midwifery education. ** Assumed to be supervised. *** Practising certificate considered to be a midwifery licence. ^a^ Qualification from the training school for nurses is considered to be equivalent to a diploma, whereas the qualification from the training school for midwives is considered to be a post-nursing qualification. Japan has numerous pathways to becoming a midwife, but this pathway was considered to be the minimum qualification required. ^b^ Primary care midwife considered here. ^c^ While the United States offers numerous midwife qualifications, only Certified Nurse–Midwives (CNM) were considered here because CNMs are licensed to practise in most areas in the United States. ^d^ General Nurse (Diploma in Nursing and Midwifery) considered here. ^e^ Rural health nurse qualification considered here. ^f^ Technical midwife qualification considered here.

**Figure A1:**
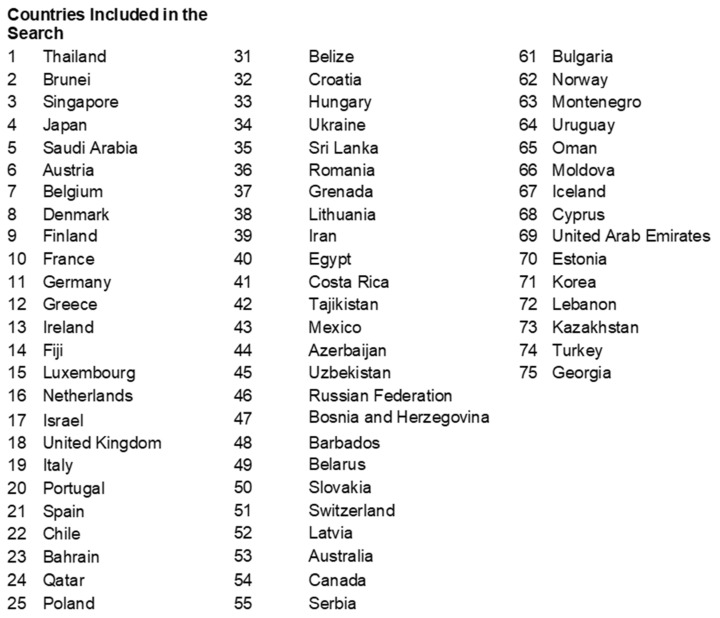
List of countries included in the study.

## Data Availability

The data presented in this study are openly available in Open Science Framework at https://osf.io/gzn3a (accessed on 5 August 2021).

## Appendix A

Search Strategy #1: Websites.

Government-related or midwifery-organisation websites from each country (Figure A1) were browsed individually for relevant documents. For websites with search bars, documents were searched for using the key word “midwives”.

Search Strategy #2: Advanced Google.

(Name of country) midwives education OR qualification OR pathway.

(Name of country) midwives licensing OR license OR registration.

(Name of country) midwives regulation OR act OR legislation OR law.

The search strings were entered into the search bar with all these words and no applied filters. Only the first two pages of results for each search string was included for screening.
